# Prostate Cancer and Neuroendocrine Differentiation: More Neuronal, Less Endocrine?

**DOI:** 10.3389/fonc.2015.00037

**Published:** 2015-03-03

**Authors:** Alexandru Dan Grigore, Eshel Ben-Jacob, Mary C. Farach-Carson

**Affiliations:** ^1^Department of BioSciences, Rice University, Houston, TX, USA; ^2^Center for Theoretical Biological Physics, Rice University, Houston, TX, USA; ^3^Sackler School of Physics and Astronomy, Tel Aviv University, Tel Aviv, Israel; ^4^Sagol School of Neuroscience, Tel Aviv University, Tel Aviv, Israel; ^5^Department of Bioengineering, Rice University, Houston, TX, USA

**Keywords:** prostate cancer, neuroendocrine differentiation, neural differentiation, interleukin-6, chromogranin A

## Abstract

Neuroendocrine differentiation (NED) marks a structural and functional feature of certain cancers, including prostate cancer (PCa), whereby the malignant tissue contains a significant proportion of cells displaying neuronal, endocrine, or mixed features. NED cells produce, and can secrete, a cocktail of mediators commonly encountered in the nervous system, which may stimulate and coordinate cancer growth. In PCa, NED appears during advanced stages, subsequent to treatment, and accompanies treatment resistance and poor prognosis. However, the term “neuroendocrine” in this context is intrinsically vague. This article seeks to provide a framework on which a unified view of NED might emerge. First, we review the mutually beneficial interplay between PCa and neural structures, mainly supported by cell biology experiments and neurological conditions. Next, we address the correlations between PCa and neural functions, as described in the literature. Based upon the integration of clinical and basic observations, we suggest that it is legitimate to seek for true neural differentiation, or neuromimicry, in cancer progression, most notably in PCa cells exhibiting what is commonly described as NED.

## Neuroendocrine Differentiation: Old Concept, Normal Counterparts, Vague Terminology

Neuroendocrine differentiation (NED) is a term referring to certain cancers that display a prominent neuroendocrine (NE) cell population on histopathologic examination. Although the definition has been used primarily in relation to prostate cancer (PCa), it is by no means restrictive [see, e.g., Ref. (1)]. NE cells display a combination of neuronal and endocrine features, best described as a partly neuron-like morphology and an endocrine-like secretory mechanism (see below). The whole NE concept itself had nothing to do with cancer; it arose in the late 1920s, when it was discovered that some hypothalamic neurons secrete their products into the bloodstream rather than into a specialized synaptic cleft as well described by Montuenga and colleagues (2). Subsequently, the existence of hybrid, neuronal-endocrine cell type, NE, was widely accepted.

Although the NED term relates to malignant tumors enriched in a NE cell fraction, NE cells are not ominous *per se*, but are part of a large cell population, collectively known as the *diffuse NE system*, which is dispersed throughout the normal organism. The NE cells primarily exist within the organs that interface with the outside world, including gastrointestinal, respiratory, and genitourinary systems, as well as the skin (Merkel cells and melanocytes). Yet, they also can be found within endocrine glands or tissues, such as the hypothalamus, anterior pituitary, pineal gland, thyroid gland (calcitonin-secreting cells), thymus, breast, and the pancreatic islets of Langerhans [reviewed in Ref. (2–4)]. For terminology clarification, we note that usually the term “NE” refers to both cancerous and non-cancer-related cells, while the term “NED” (with a “D”) refers specifically to cancerous cells. A cocktail of terms have been used interchangeably throughout the literature over the last century (e.g., APUD cells, endocrine/paracrine cells). This ambiguity arises from the visualization techniques used and the norms around the time of publication (2). This lack of standard nomenclature makes NE-related literature search particularly challenging, as some articles containing important findings can be missed.

In the healthy organism, normal NE cells play complex local regulatory roles at the tissue level. For example, the NE cells of the gastrointestinal tract (also known as enteroendocrine cells) regulate secretion, motility, as well as cell growth and differentiation in the gut. For this purpose, these cells employ endocrine, autocrine, paracrine, and neurocrine signaling mechanisms, and are, in turn, under neural control (5). The NE cells of the respiratory tract can control lung branching morphogenesis, cell growth and maturation during development, and it is believed that they provide a protective niche for a subset of lung stem cells. Similarly to enteroendocrine cells, pulmonary NE cells are under control of a complex innervation [reviewed in Ref. (4)].

## Neuroendocrine Cells in the Normal Prostate

Neuroendocrine cells are normal inhabitants of the human prostate, existing in all areas of the gland, including prostate ducts, acinar epithelium, and prostatic urothelium, but they localize preferentially in the major ducts [reviewed in Ref. (3, 6)]. Prostatic NE cells are found in lower numbers in African-American males, who are more prone to developing PCa; NE cells thus might have a protective role against prostatic carcinogenesis (7). As with all NE cells, the NE prostatic cells usually cannot be recognized under the light microscope using conventional staining techniques, but can be readily traced immunohistochemically by staining for the specific markers chromogranin A (CgA), synaptophysin, or neuron-specific enolase (NSE). In some cases, one or more NE markers may be absent [reviewed in Ref. (8)].

Prostatic NE cells share the morphological and ultrastructural features of NE cells from other parts of the body [reviewed in-depth in Ref. (2); also reviewed in Ref. (4, 5)]. Under the electron microscope, two different morphologies were described. The *open-type* cells display thin apical processes that extend luminally, reach the lumen, and possess long surface microvilli. The *closed-type* cells have dendritic-like processes that extend between adjacent epithelial cells, but do not reach the lumen. The closed-type cells are surrounded by epithelial cells. Although no study has specifically addressed this question for the prostate, it is assumed that this morphological classification also has an important functional significance. Closed cells can only receive basal stimuli (neurotransmitters from nerve endings, hormones from neighboring blood vessels, local paracrine, or autocrine factors from underlying stromal cells). By contrast, open cells also can receive luminal stimuli (pH, chemicals). It is therefore generally believed that the open and closed NE cell populations, irrespective of their specific location, are functionally different [reviewed in Ref. (2)].

The NE cells of the prostate contain secretory granules whose electron microscope features allow further classification [reviewed in Ref. (3, 8)]. The contents of the secretory granules display a remarkable diversity and belong to the family of neuromediators that are used for signaling throughout the nervous system. Apart from three NE markers CgA, synaptophysin and NSE, NE cells synthesize other members of the chromogranin family as well as a variety of hormone-related substances, including chromogranin B and chromogranin C (secretogranin II); serotonin; histamine; thyroid-stimulating hormone-like peptide; calcitonin and related peptides (calcitonin gene-related peptide, katacalcin); α-human chorionic gonadotropin; somatostatin; bombesin; parathyroid hormone-related protein; vasoactive intestinal peptide; neuropeptide Y; cholecystokinin; vascular endothelial growth factor (VEGF); glucagon; β-endorphin; Leu-enkephalin; and adrenomedullin [reviewed in Ref. (2, 3, 6, 8, 9)]. It remains unclear if a single NE prostatic cell can synthesize this huge cocktail, or a vast majority, or only a subset thereof. Regarding the diffuse NE system, it is known that, in principle, single NE cells can produce more than one hormone-related substance [reviewed in Ref. (2)]. Some prostatic NE cells were reported to produce two mediators instead of one, and it is apparent that several subpopulations of NE cells exist in the prostate, each of them producing a specific subset of mediators (10). Receptors for some of these neuromediators were described in benign prostatic tissue and/or in PCa and include receptors for serotonin, calcitonin, bombesin, somatostatin, cholecystokinin, neuropeptide Y, and neurotensin [Ref. (11, 12); see also Ref. (8) and the references therein].

Although NE cells were first described in the normal prostate 60 years ago [Grasso, 1954, cited in Ref. (13)], few studies have addressed their function. Do normal prostatic NE cells actually secrete all those compounds they synthesize? Do they regulate other cells and if they do, then what are the regulatory mechanisms? What exact role(s) does each of those compounds have in the prostate, if secreted? These are all questions that remain to be addressed, as most of the data available come from extrapolation. For example, CgA, which is one of the most prominent NE markers, regulates the secretory vesicle pool and calcium homeostasis, and it accompanies catecholamines in the secretory vesicles in the sympathetic and adrenomedullary systems [reviewed in Ref. (14)], but there is considerably less evidence as to its specific roles in the prostate. Similarly, the roles of the other neuropeptides are incompletely understood. However, the neuropeptides influence depolarization, modulate ionic currents, release calcium from intracellular stores, stimulate ATP synthesis, stimulate oxidative phosphorylation, and regulate mRNA transcription. Globally, prostatic NE cells are thought to play a key role in prostate growth and differentiation [reviewed in Ref. (15)].

In early descriptions, prostatic NE cells displayed heterogeneous cytokeratin expression (a classification into basal, luminal, and intermediate NE cell types is based on this criterion) [reviewed in Ref. (16)]. More recent accounts indicate that prostatic NE cells express K5 cytokeratin, which is a basal cell marker [reviewed in Ref. (8)]. NE cells appear to be non-proliferative, postmitotic, as they lack the proliferation marker Ki-67. They, however, lie preferentially adjacent to proliferating and Bcl-2-positive cells, a pattern suggesting that NE cells support the growth of non-NE cells through paracrine mechanisms [Ref. (15, 17, 18); also reviewed in Ref. (2)]. However, most proliferating non-NE cells do not lie close to NE cells (17), which makes this relationship harder to rationalize. Another prominent feature of these cells is the lack of androgen receptor (AR) [Ref. (19); also see Ref. (6, 16, 20) and the references therein]. This is particularly intriguing, as androgens are considered to be the most important growth-supporting factor in the prostate, with innervation being the second most important (21). In animal models, NE cell number and morphology are not influenced by castration or prostatic denervation (22). In fact, it remains unclear which factors account for the regulatory control of NE prostatic cells, or if these regulatory signals are endocrine, paracrine, autocrine, neurocrine, or “lumencrine” (i.e., signals in the duct lumen itself) (23).

The developmental origin of these cells long has been a matter of debate. Normal prostatic NE cells likely share a common developmental origin with urogenital sinus-derived luminal and basal cells. A second lineage was identified that has a neurogenic origin from periprostatic paraganglia (24). Consequently, some authors have proposed that these cells have a neurogenic origin, arising from the paraganglia that flank the urogenital mesenchyme; by subsequent migration, these precursor cells populate the prostatic epithelium. Other authors have suggested that prostatic NE cells have a local prostatic origin, arising through differentiation of a local pluripotent stem cell that gives rise to all the epithelial cell types in the prostate [Ref. (20); also reviewed in Ref. (2)]. Recent work leans to the local prostatic origin hypothesis. During development, postnatal development, as well as in the adult organism, the prostatic NE, luminal, and basal cells arise through differentiation of local multipotent stem cells expressing the p63 protein marker (25–27).

## Neuroendocrine Differentiation in Prostate Cancer: Separate Categories vs. Continuum

**Figure 1:**
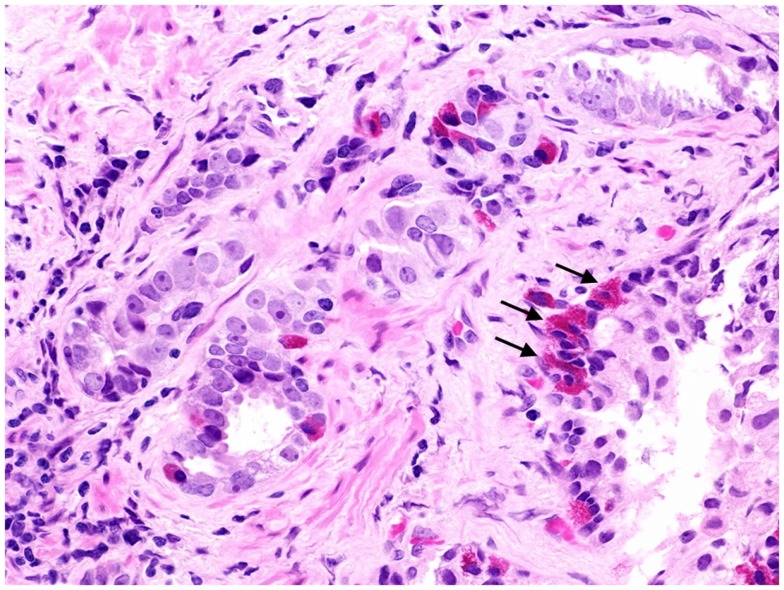
**Prostate cancer displaying focal neuroendocrine differentiation**. Focal NED typically requires specific staining methods. However, in about 10% of cases, NE cells display large eosinophilic granules recognizable by conventional staining (arrows). In focal NED, the NE cells occur either as solitary cells or in clusters. H&E stain. Courtesy and with permission of Dharam M. Ramnani, MD; WebPathology.com.

**Figure 2:**
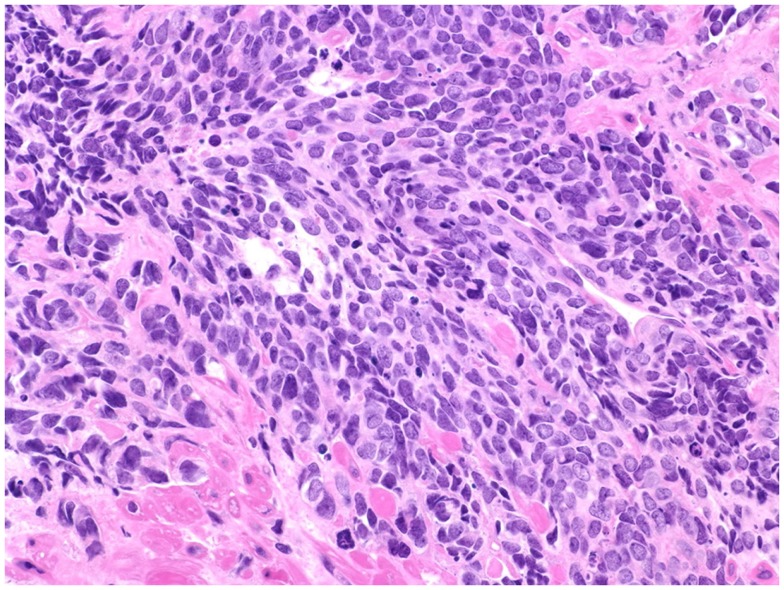
**Prostate cancer displaying universal neuroendocrine differentiation**. Universal NED is synonymous to SCCP. This cancer type is rarer than conventional prostatic adenocarcinoma (fewer than 1% of total PCa cases) and prognosis is dismal. Histologically, cells display scarce cytoplasm, hyperchromatic nuclei with finely dispersed chromatin and inconspicuous nucleoli, and nuclear molding. Mitotic index is high and necrosis often is present. In about half of the cases, the small cell carcinoma is admixed with areas of conventional prostatic adenocarcinoma. The Gleason scale cannot be used for pure SCCP, but in mixed cases it should be used to grade the adenocarcinoma regions. H&E stain. Courtesy and with permission of Dharam M. Ramnani, MD; WebPathology.com.

In the context of prostatic malignancy, NED is a highly heterogeneous phenomenon. From a spatial viewpoint, there are (i) tumors that are purely NE, such as small cell carcinoma of the prostate (SCCP), carcinoid, and carcinoid-like tumors, or (ii) tumors that are non-NE (e.g., adenocarcinomas) but exhibit rather focal NE features (in primary and/or metastatic sites). These can be further divided with respect to timing: some adenocarcinomas display large populations of NE cells from the start, while others recur as NE carcinoma later on [reviewed in Ref. (3, 28)]. Some points of this categorization remain debatable [e.g., more recent studies of NED have deliberately excluded carcinoid and carcinoid-like tumors as belonging to a different histological category, while others (29) have considered them as NED tumors]. The classification, however, emphasizes two important elements. First, the *extent* of NED varies across patients, in that some tumors exhibit *focal NED* (i.e., only a subpopulation of tumor cells exhibit NE features) (Figure 1) while others display *universal (pure) NED* (i.e., the tumor is entirely composed of NE cells). The universal NED is, in fact, SCCP, which accounts for 1% of the prostatic malignancies and, similarly to small cell carcinomas from other organ sites, has a particularly poor prognosis (30) (Figure 2). Second, the *timing* of NED varies across patients, in that certain patients show NED tumors from the beginning, while others receive treatment for conventional PCa and later experience recurrence with NED tumors.

An important question to be asked is, therefore, if the NED categories described above are discrete phenotypes or if they represent a continuum of phenotypes. First, it is known that some histological and immunohistochemical traits are common to conventional prostatic adenocarcinoma and SCCP (31). Second, there is a growing body of literature reporting therapy-associated progression from (i) conventional prostatic adenocarcinoma to focal NED, (ii) focal NED to SCCP, or (iii) conventional prostatic adenocarcinoma to SCCP (32–40). Progression to these NED categories occurred in any stage of the disease (i.e., organ-confined, locally advanced, metastatic) (32–34, 36–39). Third, increasing evidence shows cases of mixed adenocarcinoma/SCCP tumors. In these patients, disease stage correlates directly, while survival correlates inversely, with the proportion of the SCCP fraction and the grade of the associated adenocarcinoma fraction (30), suggesting that PCa gains in relative SCCP proportion as the disease progresses. SCCP might, in fact, represent the least differentiated type of conventional prostate adenocarcinoma (i.e., beyond the Gleason 10 score), which would indicate adenocarcinoma and SCCP form a continuum (30).

**Figure 3:**
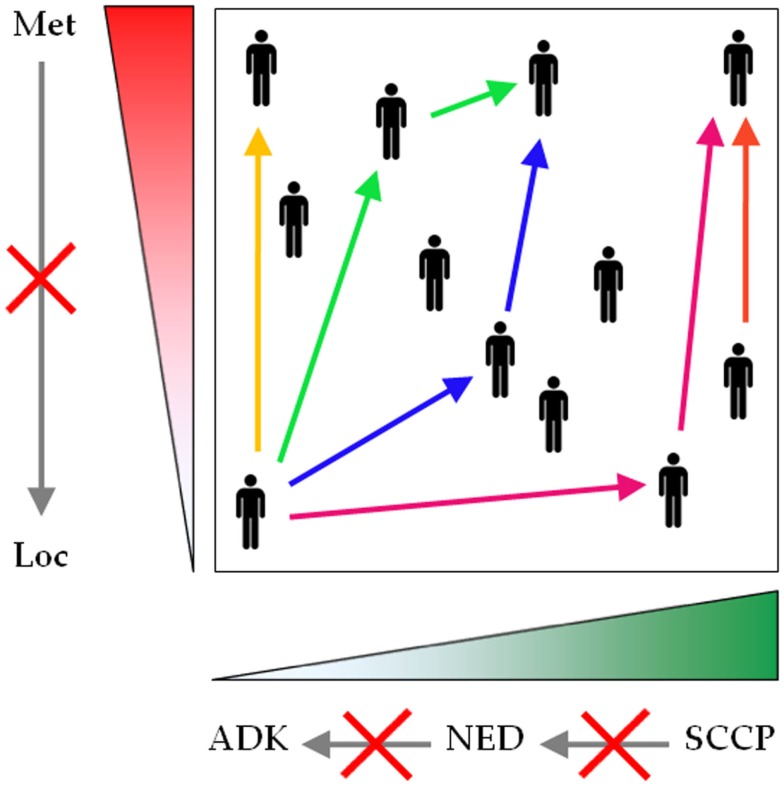
**Neuroendocrine differentiation spans a continuous and dynamic range**. Conventional prostatic adenocarcinoma, focal NED, and SCCP traditionally are represented as separate entities, but evidence shows that they rather form a unique spectrum of prostatic malignancies. The common denominator encompasses two parameters: the extent of NED (green gradient), spanning from zero (conventional prostatic adenocarcinoma) to universal NED (SCCP), and the extent of malignant dissemination (red gradient), ranging from localized to metastatic disease. During first presentation or reoccurrence, NED can be diagnosed at any point across this plane (black human icons). For any patient, any parameter can shift to a higher value at any stage during the course of the disease (see color-coded arrows, each color corresponding to one patient), but no parameter shifts to a lower value (gray arrows crossed by red saltires). Loc, localized disease; Met, metastatic disease; ADK, conventional prostatic adenocarcinoma; NED, focal NED; SCCP, small cell carcinoma of the prostate.

Collectively, these data strongly suggest that (i) NED in PCa is a dynamic and continuous range, spanning from conventional adenocarcinoma to SCCP; (ii) progression across this range is directed from conventional adenocarcinoma to SCCP; (iii) progression can occur at any stage of the disease; and (iv) progression is driven by therapy (Figure 3).

Last, it is worth mentioning that a new classification of NED has been proposed recently (29), in which the extent and timing of NED have been abandoned and NED is categorized solely on morphologic observations. As the authors themselves acknowledge, apart from the purely histopathologic perspective, most of the new categories proposed bear little clinical significance (29). Based on the existing literature discussed above, we suggest that the present classification should be kept in place, and refined only as the full genotypic and phenotypic character of NED is discovered.

## Neuroendocrine Cells in Prostate Cancer

Neuroendocrine cells first were reported in malignant prostate tissue almost 45 years ago (41), making it the first description of NED in PCa. Similarly to normal NE cells, the NE tumor cells express various neuromediators (see below).

It is important to unravel the phenotypic and genotypic associations between normal and tumor-associated NE cells. Surprisingly, NE tumor cells are in many ways indistinguishable from their adjacent, conventional non-NE tumor counterparts (42). First, NE tumor cells express K18 and K8 cytokeratins, which also are expressed by luminal cells of the prostatic epithelium and also by conventional tumor cells (i.e., adenocarcinoma cells). However, they do not express high-molecular weight cytokeratin and p63, which are associated to the basal cells of the prostatic epithelium (43, 44). By contrast, normal NE cells mainly express K5, a basal cell marker (see above). Second, both NE tumor cells and non-NE tumor cells express the β-oxidation enzyme α-methylacyl-CoA-racemase (AMACR) (43), a recently identified marker that is strongly associated with PCa risk (45). By contrast, normal NE cells lack this marker (43). Third, genetic analysis revealed that NE tumor cells are similar to non-NE tumor cells rather than to NE normal cells. The NE normal cells, in turn, are similar to the non-NE normal cells (46). It is thus apparent that belonging to the malignant vs. benign prostatic phenotype is a stronger clustering factor than belonging to the NE vs. non-NE cell type.

Similarly to NE cells in the normal and hyperplastic prostate tissue, NE tumor cells are non-proliferative, postmitotic cells (17, 18, 43, 44), and they lack stem-like cell markers ALDH1, NANOG, and CD44 (44). They are mainly adjacent to proliferating (17, 43) and Bcl-2-positive cells [see Ref. (16) and the references therein], and such NED areas exhibit the highest proliferation index across the tumor tissue (47). It is therefore not surprising that, the more NED areas a tumor displays, the higher the proliferation index at the global tumor level; that is, the extent of NED correlates with higher proliferation index of the whole tumor [Ref. (47), although see Ref. (17)]. The NE tumor cell density per NED area further enhances proliferation. Proliferation index among NE cells is higher in tumors displaying clusters of NE cells as compared to tumors displaying solitary NE cells or no NE cells (47).

How are NE tumor cell signals broadcast, though? In mice, androgen-dependent LNCaP prostate tumors can grow in castrate conditions only in the presence of NE tumors, which suggests that NE tumor cells secrete some long-range, endocrine factors (48). However, the effect is not seen *in vitro*, as conditioned medium from NE tumor cells does not rescue decreased growth of LNCaP cells in androgen-depleted conditions (48). Although this difference between *in vivo* and *in vitro* could be attributed to a different gene expression profile of LNCaP and/or NE cells, it also could be accounted for by the absence of NE cells in the *in vitro* experiments. Namely, it is possible that short-range, paracrine factors [as hypothesized more than two decades ago (17)], or direct cell–cell contact are responsible for the NE tumor cell supportive role. Indeed, LNCaP cells proliferate more than twofold faster when co-cultured with LNCaP cells displaying NED (49), which further supports this view.

It is commonly accepted that NE tumor cells are AR- and PSA-negative and prostatic acid phosphatase-positive [Ref. (43, 44), see also Ref. (50) and the references therein]. Older studies suggest that a small minority of NE tumor cells display some AR expression (19). The lack of AR makes them androgen-independent, as are NE normal cells. In an *in vivo* model of human PCa xenograft subjected to androgen deprivation (castration), the residual tumor is enriched in NE tumor cells, which appear to result by selection, i.e., survival, despite the lack of androgens (44).

Regarding the origin of NE tumor cells, it has been suggested that PCa NE cells share a common intermediate stem cell origin with their normal NE counterparts. Others hypothesized that since normal NE cells do not proliferate, PCa NE cells are likely to arise through transdifferentiation from either conventional prostatic adenocarcinoma cells or prostatic exocrine cells becoming malignant [Ref. (18); also reviewed in Ref. (8)]. The stronger genetic clustering of tumor NE/non-NE cells as compared to tumor NE/normal NE cells lends further support to the second hypothesis (46).

Interestingly, not all NE cells found in PCa are genuine NE tumor cells. Sion-Vardy’s group described a CgA- and serotonin-expressing NE cell population in the normal peritumoral regions (10). By contrast, other NE markers (NSE and adrenomedullin) were expressed uniformly across normal peritumoral and tumoral regions, and across PCa and benign prostate hyperplasia patients, respectively, which suggests that the prostate may contain several different NE cell populations (10). It might be that PCa induces neighboring benign cells to transdifferentiate into NE cells, which then promotes tumor growth through their secretory products (10). Neuropeptides of NE cells promote tumor growth *in vitro* (51), but the functions of various mediators have only begun to be unraveled.

It is particularly intriguing that not all tumor-produced neuromediators are uniformly tumor-supportive. For instance, CgA, which is among the most prominent NED markers, displays competing activities. CgA is active in its full-length form, but it is also physiologically cleaved at various sites, generating about a dozen of bioactive fragments [reviewed in Ref. (14)]. These hormones play intricate regulatory roles in vascular and tumoral biology, either as pro-angiogenic or anti-angiogenic factors [reviewed in Ref. (52)]. Vasostatin-1, one of the most widely studied CgA fragments, inhibits tumor angiogenesis, apparently by inhibiting the endothelium-stimulatory effects of hypoxia and tumor-secreted factors. This, in turn, precludes the activation of endothelial cells and preserves the integrity of the endothelial barrier. At the molecular scale, vasostatin-1 inhibits hypoxia-driven nuclear translocation of HIF-1α (53). By contrast, full-length CgA has a biphasic effect. While physiologic concentrations inhibit both spontaneous and VEGF- and FGF-2-induced angiogenesis, the anti-angiogenic effect is lost at supraphysiologic CgA concentrations, suggesting that high CgA levels, as occur in NE and some non-NE cancers, might reduce the anti-angiogenic effect (54). Moreover, thrombin provides an angiogenic switch characterized by gradual cleavage-induced inactivation of anti-angiogenic fragments of CgA, coupled to cleavage-induced generation of pro-angiogenic fragments of CgA. This switch is relevant in clinical conditions exhibiting thrombin activation, including cancer (54), a hallmark of which is angiogenesis [reviewed in Ref. (55, 56)]. CgA and CgA-derived hormones also play opposing roles in regulating tumor proliferation. In mice, CgA inhibits transit of mammary cancer cells among primary tumor, blood, and organ compartments (57). Mechanistically, CgA inhibits tumor cell-induced formation of endothelial gaps and decreases TNFα-induced vascular leakage (58), reduces vascular leakage within tumors, and inhibits tumor cell transendothelial migration (57). However, vasostatin-1 and -2 stimulate proliferation in small intestinal NE metastatic cell lines via Akt phosphorylation, although they do not affect small intestinal NE primary tumor cell lines (59). In PCa cell lines, various CgA fragments have opposing roles, as some stimulate, while others inhibit invasion, haptotactic migration, and growth [see Ref. (60) and the references therein]. It becomes thus apparent that factors produced by NE tumors can exert effects of both polarities, either enhancing or diminishing tumor development.

## Inducers of Neuroendocrine Differentiation

Numerous molecular signals and pathways connect to NED, or to functional features commonly associated with it, such as androgen-independent growth. These consist of (i) ligands that induce NED or NED-related features, and (ii) signals generated by NE cells that affect tumor dynamics. The distinction between the two categories is blurry, as some of the latter stimuli can themselves induce NED. The entire repertoire of NED inducing factors include neuromediators (bombesin, calcitonin, serotonin, and vasoactive intestinal peptide) (11, 51, 61, 62), cytokines (IL-1β, IL-6, and IL-8) (11, 63–75), ionizing radiation (76), elevated intracellular cAMP [Ref. (77); also see Ref. (46) and references therein], Wnt proteins (78), PI3K–Akt–mTOR pathway (79), and high-cell density (80). NED also can be at least partially reversed, and the extent of reversibility depends on the NED inducer (46, 76).

While NED transition can be induced by various cues, it mainly results from androgen deprivation therapy (ADT) [Ref. (81); also see cases documented in Ref. (32–36, 38, 39)], which is done by pharmacological or surgical castration and is the standard of care in advanced PCa (82). Because virtually all PCa cells rely on androgens to grow, ADT can hold the disease at bay for a while, thus increasing progression-free survival. However, nearly all patients on ADT eventually develop androgen resistance (82), a term that, although extensively used in the literature, is somewhat confusing. The PCa cells develop the ability to grow in the absence of androgens, but it is the androgen deprivation they become resistant to, not the androgens themselves. Clearly stated, PCa cells become androgen-independent, i.e., self-sufficient to androgen growth signals, or ADT-resistant, which is a first hallmark of cancer (55, 56). In this text, we will use the synonymous term: castrate resistance (CR).

In PCa, more aggressive ADT promotes more rapid NED transitions [reviewed in Ref. (83)]. In mice, androgen depletion triggers regression and NED within the primary tumor. The androgen depletion-induced NED is proliferation-independent, and NE tumor cells exhibit increased expression of serotonin, bombesin, and somatostatin (16, 44). In patients with metastatic PCa, serum CgA levels are associated with duration of ADT (84). Moreover, CgA levels increased faster in patients with PSA failure than in patients without it, suggesting that velocity of CgA increase might help predict the risk of biochemical failure after ADT (84). The rhythm of ADT administration might also play an important role in NED dynamics. Sciarra’s group found that continuous ADT significantly increased serum CgA in both localized and metastatic PCa patients, whereas intermittent ADT did not influence serum CgA in either patient subset (85). Interestingly, AR expression did not correlate with presence of NE cells or with biochemical recurrence, which is consistent with previous observations and suggests that NE cells do not influence AR expression in neighboring cancer cells (86).

Because ADT promotes both CR and NED, an important question is if CR and NED go hand-in-hand and are causally linked? Observations published to date make it hard to give a definite answer. However, among patients who develop NED following ADT, more than 80% developed CR at an intermediate point between ADT initiation and NED (40), so at least a subset of patients with CR will develop NED during their clinical course (87). Although irrefutable evidence is still lacking, it is therefore usually implied that the most common clinical sequence leading to NED is:

Advanced conventional prostatic adenocarcinoma→ADT initiation→CR→NED

It should be kept in mind, however, that the alternative sequence:

Advanced PCa displaying NED→ADT initiation→CR,

in which NED precedes CR, also is encountered in the clinic. If found in treatment-naïve patients, NED may predict a poor response to ADT (see next section), in which case NED must have occurred first. Moreover, as NED can develop at any stage of the disease (Figure 3), still other clinical sequences also are possible.

## Clinical Significance of Neuroendocrine Differentiation in Prostate Cancer

In PCa patients, NED is a frequent histopathological finding, ranging from 31 to 100% of cases in primary tumors (9, 17, 42, 47, 86, 88–92), while occurring in 12% of metastatic lymph node samples (90). It has been proposed that, in PCa, NED cells appear in tissue regions that are similar to non-cancerous atrophic glands (86). The most frequently expressed neuropeptide across tumor samples is calcitonin (37.1% of samples), followed by neurotensin (11.4%), serotonin (10%), α-human chorionic gonadotropin (8.6%), vasoactive intestinal peptide (5.7%), and bombesin (2.9%) (86). Using less restrictive criteria, others reported NSE (77% of the samples) and CgA (59%) as the most frequently expressed neuropeptides (9). However, significant variations were found in these studies with respect to the NED markers used and the internal structure of the patient groups (most notably, the clinical setting and the prior therapy). We note that while all these immunohistochemical markers might be useful for diagnosing NED cancers, it is unclear if they also are useful for new therapeutic strategies. Developing new therapies requires assessing how efficiently a potential drug reaches its target. In addition, since NED most often is a focal process, histopathologic markers are less accurate than serum markers. On the other hand, serum markers are expressed by prostatic non-NE cells as well; hence, their levels depend on global prostatic tissue volume rather than on specific NE cell number (93).

This chapter mainly discusses NED from a histopathological, rather than a functional, standpoint. Therefore, we will follow the nomenclature tradition of the existing body of literature. Histopathologically, while focal NED is relatively frequent, universal NED is a rare event (accounting for 1% of prostatic malignancies). For this reason, focal NED is commonly referred to as simply “NED,” while universal NED is usually referred to by its well-known pathological term “SCCP.” For consistency, we will follow this convention.

### SCCP (universal NED) and prognosis

**Figure 4:**
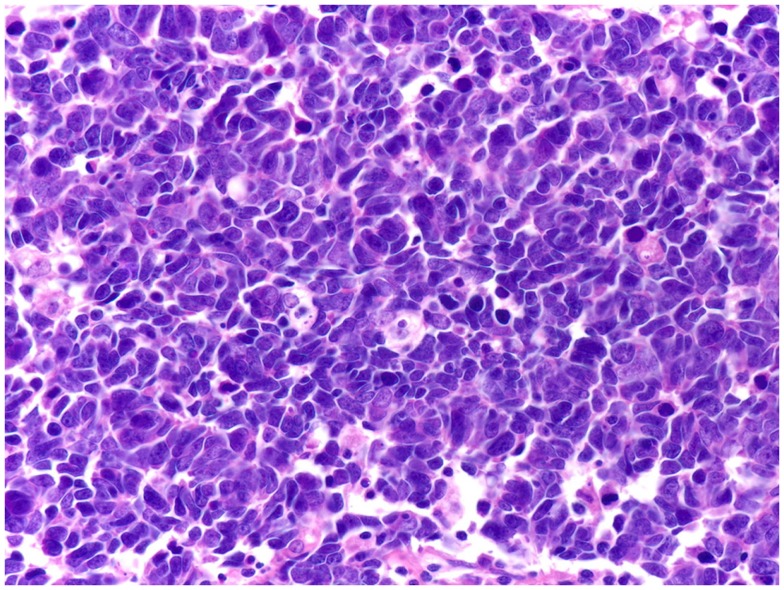
**Small cell carcinomas are a large, homogeneous family of cancers**. The histology, therapeutic regimens, response to therapy, and prognosis of SCCP are strikingly similar to small cell carcinomas of all the other organ sites, including SCLC shown here. Extensive nuclear molding and apoptotic bodies can be seen. See Figure 2 for comparison with SCCP. H&E stain. Courtesy and with permission of Dharam M. Ramnani, MD; WebPathology.com.

Conventional prostatic adenocarcinoma, which accounts for the vast majority of PCa cases, has an excellent prognosis due to its slow clinical course and the available screening tests. By contrast, SCCP is a fatal disease. In patients with SCCP, 2- and 5-year survival rates are 27.5 and 14.3%, respectively, while median survival is 15 months for locoregional disease and 7 months for metastatic disease (30). Histopathologically, SCCP belongs to a large family of cancers referred to as *small cell carcinomas*, which share identical tissue architecture (small cells, round- or spindle-shaped, displaying sparse cytoplasm, nuclear molding, high-mitotic index, and frequent necrosis) that can be readily identified by conventional hematoxylin and eosin staining [reviewed in Ref. (94–96)]. According to the site of origin, small cell carcinomas are conventionally divided into *small cell lung carcinoma (SCLC)*, the most frequent, and *extrapulmonary small cell carcinomas (EPSCC)*, which include cancers from all other sites, including SCCP. Although rare, EPSCCs have been documented in virtually all organs in the body (94, 97–99), including the brain, which had traditionally been considered not to display primary SCC tumors (100). Despite this diversity of organ sites, EPSCC and SCLC exhibit virtually identical patterns of behaviors: extremely poor prognosis, quasi-identical therapeutic regimens, high- and short-lived initial response to platinum agents, and topoisomerase inhibitors, followed rapidly by tumor relapse and death, shared molecular alterations, and similar histopathology (Figure 4). This clinical, pathological, therapeutical, and prognostic pattern for both EPSCC and SCLC has remained unchanged over the last decades (94, 96, 98, 99, 101–106), and the only organ sites where EPSCC displayed better prognosis are breast (99, 104) and female reproductive tract (104), most notably the cervix (102, 105). However, the prognosis for breast SCC is poorer as compared to all other types of non-inflammatory breast cancers combined (107). Likewise, the prognosis of cervical SCC is poorer than the prognosis for squamous cell carcinoma and adenocarcinoma of the cervix, respectively (105). Thus, even for these situations where EPSCC breaks the rule and displays better outcomes, the relative prognosis is poorer compared to other cancers originating from the same site, and the pattern is therefore preserved. Collectively, these data strongly suggest that universal NED is a unique disease that displays a consistently homogeneous pattern of tumor behavior and clinical outcomes irrespective of the organ site of origin.

### NED (focal NED) and prognosis

After two decades of research, it is commonly perceived that NED in PCa indicates poor prognosis. This impression could be due to a mere association, as NED correlates with tumor grade [reviewed in Ref. (3); see below]. Thus, it might not be NED itself, but the high tumor grade it accompanies, which accounts for the poor prognosis. Alternatively, this could be a true causative relationship, in which the NE tumor cells themselves generate a resistance to hormonal therapy. This idea is consistent with repeated findings that PCa that exhibit NED are hormone-resistant [reviewed in Ref. (3); also see below], as well as with the fact that adenocarcinomas tend to recur after hormonal therapy as carcinomas with focal NED [reviewed in Ref. (3)]. It is worth noting that a correlation between NED and poor prognosis would be encountered in both scenarios (NED as merely associated with, vs. NED as causatively related to, poor prognosis). This correlation is likely to have a strong impact on a grand scale, because PCa is the most common cancer and the second-leading cause of cancer death in American men (108). Thus, even though patients diagnosed with advanced PCa represent only a small fraction of the total number of PCa cases, they still represent a high absolute number. Consequently, the ADT-induced NED, which is encountered in these patients, also is expected to occur in a high absolute number of patients.

It is difficult to get an accurate picture of the association between NED and prognosis, mainly because of the lack of large, conclusive studies. Instead, many studies report that NED correlates with poor prognosis, and still other studies fail to report such association. The main reason is the methodological heterogeneity in common practice to detect and quantify NED, to collect biological material, and to devise patient inclusion criteria [for a snapshot see Ref. (40)]. This heterogeneity is threefold.

**Figure 5:**
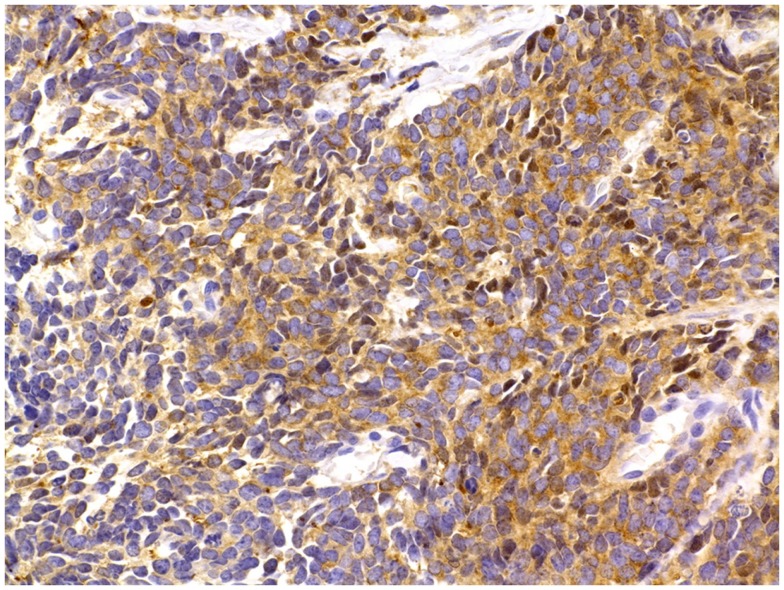
**Prostate cancer immunostaining for neuroendocrine markers**. In surgical or core needle biopsy samples, NED can be detected by immunostaining against specific markers, most notably peptides, which are present inside the NE secretory vesicles (see text for details). A frequently used NED marker is the enzyme NSE, seen here as brown cytoplasmic granules. Immunoperoxidase stain. Courtesy and with permission of Dharam M. Ramnani, MD; WebPathology.com.

First, NE cells produce a vast array of neuropeptides that are used somewhat stochastically in immunostaining procedures to identify NED. However, the freedom to choose among various markers relies on the assumption that NED markers are equivalent to one another, i.e., they are present in the tissue in equal or proportional quantities. But is this assumption true? The most recent explicit methodological directive implies that any of the three common markers (CgA, NSE, or synaptophysin) is sufficient to document NED (8) (Figure 5). In patients with either localized or advanced (stage D2) PCa, strong CgA staining of primary tumor correlates with poorer cause-specific survival (109, 110) and overall survival (90) and provides superior information as compared to currently used pathologic prognostic factors (109). Similarly, CgA abundance in lymph node deposit also correlates with poorer overall survival (90). By contrast, NSE staining of primary tumor does not correlate with survival (110). In fact, as stated above, NSE was equally expressed in tumoral, peritumoral, and benign prostatic hyperplasia (BPH) tissue (10), which suggests that it lacks clinical significance. Thus, although not systematically demonstrated, CgA is the most clinically relevant NED marker (91) and has been used in most studies addressing the clinical implications of NED.

Second, tumor samples can be obtained either through a surgical intervention or a core needle biopsy. However, the freedom to choose between these two procedures relies on the assumption that they are equivalent procedures. But are they? At a first glance, while surgery ensures pathological sampling access to the whole tumor, biopsy is like looking through the keyhole. It is thus unclear if NED seen in the biopsy accurately mirrors the NED of the whole tumor. In tumor biopsies from patients with advanced PCa (stage D2), strong CgA immunostaining was correlated with higher 2-year recurrence rates and much shorter time to recurrence (111). Moreover, in biopsies from an unselected population of patients with PCa, biopsy CgA immunostaining positivity correlated with shorter survival (112). Similarly, in biopsies of a selected population of patients with ADT-resistant PCa, NED correlated with decreased cancer-specific survival (113). Additionally, in core needle biopsies from PCa patients with Gleason score 8–10 who received primary radiotherapy, CgA immunostaining >1% correlated with less favorable biochemical control, clinical failure, distant metastases (sixfold), and cancer-specific survival (almost fivefold) rates as compared to CgA immunostaining <1% or negative CgA immunostaining, respectively (92). Other authors, however, after assessing biopsies from patients with ADT-resistant PCa, reported no significant correlation between CgA serum levels and biopsy CgA immunostaining intensity (12). Because CgA serum levels correlate with CgA immunostaining in the primary tumor (see below), biopsy tissues may provide a distorted, inaccurate image of NED. To avoid this gray zone of uncertainty, a best approach might be to use biopsy CgA immunostaining in tandem with CgA serum levels to increase overall accuracy (114). Although, in most cases, biopsy appears to provide a fair image of the extent of NED in the primary tumor, it should be remembered that surgical samples provide the most reliable snapshots of NED.

Third, a majority of the neuropeptides can not only be viewed immunohistochemically but also can be measured in serum. However, the freedom to choose between these two methods relies on two assumptions: (i) NED markers produced by NED cells are released into the bloodstream, and (ii) there is a linear relationship between NED in the tissue and NED markers in the blood. But are these two assumptions true? While obtained more easily and less invasively, serum levels provide an indirect (and perhaps distorted) measure of the NED presence and extent. In addition, blood levels of several NED markers can increase in non-NED settings which thereby act as confounders (e.g., chronic use of proton pump inhibitors increases CgA levels nearly eightfold) (115). However, CgA serum levels do correlate with CgA staining of primary tumor, and they also correlate with CgA staining of biopsy samples (9, 110, 114), which suggests that serum CgA accurately mirrors the extent of NED in PCa. By contrast, no correlations exist between immunohistochemical staining for chromogranin B, NSE, and pancreastatin, respectively, and the corresponding serum levels of those markers (9, 110). Thus, CgA is the most reliable serum marker of NED (9). CgA serum levels are higher in PCa as compared to BPH, and they are higher in BPH as compared to healthy controls (116). In patients with ADT-resistant PCa, high serum levels of CgA correlate with reduced overall survival (12). In patients with ADT-resistant PCa who had not received any prior chemotherapy, CgA serum levels increased rapidly over time (twofold in 9 months), correlate negatively with survival, and are an independent predictor of survival (117, 118). However, this might only apply for a subset of patients. Two groups showed that, in patients with advanced PCa, elevated serum CgA only correlates with poor prognosis when serum PSA is below or equal to the median value, but fails to correlate when serum PSA is above the median value (117, 119). The relationship between serum NSE levels and prognosis is less clear-cut, but positive correlations have been reported. NSE serum levels are higher in PCa as compared to BPH and they are higher in BPH than in healthy controls (116). In patients with advanced PCa, elevated pre-treatment serum levels of NSE correlate with short survival (120). However, in patients with advanced PCa, pre-treatment serum levels of NSE do not correlate with those of CgA, which correlate with NED and prognosis. Moreover, following palliative radiotherapy, serum levels of NSE drop while serum levels of CgA and PSA increase. This suggests that CgA- and NSE-secreting NE cells differ from each other in terms of radiosensitivity (120). The authors rely on this finding to suggest that NSE, rather than providing prognostic information, might actually help monitor the response to palliative radiotherapy (120).

Associations between NED and other tumor features also are linked to prognosis. Quek’s group found that NED in the primary tumor correlates with clinical recurrence (90). Others reported that NED correlates with high Gleason score (88, 89, 114, 116, 121), tumor stage (89, 112, 114, 121), and the presence of metastases (89). Pattern of NE cell growth also is important. Tumors with high Gleason score (7 to 10) tend to show clusters of NE cells, while most tumors with low Gleason score display solitary NE cells, and presence of clusters appears to impart a poorer prognosis (47). NED also correlates with serum PSA levels, but conflicting evidence exists regarding the directness of the correlations (114, 121). Similar observations, with less specific NE markers, were made by Ishida’s group. In tumor samples from patients who had not received preoperative therapy, the presence of calcitonin correlated with high Gleason score, suggesting an association with cancer aggressiveness or invasion (86). Furthermore, in PCa patients, serum levels of CgA and NSE correlated with tumor stage, with a slightly higher resolution for CgA (116).

Not all the studies have found associations between NED and clinical, biochemical, or pathological features of PCa. Various authors failed to report any correlation between NED and prognosis, failure after radical prostatectomy, clinical stage, Gleason score, tumor stage, or lymph node metastasis (42, 88, 122). Others reported no correlation between NED and proliferation index or disease progression (42), or between NED and PSA levels or PSA progression (89, 112). Among the most widely used NED markers, NSE serum levels failed to correlate with Gleason score (116). Some of these studies, however, did not properly explore the correlations between NED and survival or between NED and more specific subsets of patients (e.g., those receiving ADT or displaying CR) (42, 122). This is precisely why these null results are important. That is, if corroborated with the correlations described before, they suggest that NED might rather predict a poor response to ADT than a globally poor prognosis (42, 112). Indeed, in PCa biopsies from patients with newly diagnosed PCa who subsequently received ADT, high CgA immunostaining correlated with faster progression to CR and lower overall survival; moreover, in these patients, CgA serum levels almost doubled after 2 years of ADT (114). This increase in CgA serum levels during ADT might reflect the development of increased tumor aggressiveness and CR (114). Furthermore, although biopsy CgA immunostaining does not correlate with shorter time to PSA progression in patients not treated by ADT, it does correlate in those receiving ADT (112). The correlation between NED and prognosis might therefore need to be stratified further across patient subsets, most notably those receiving ADT. In addition, a methodological systematization with respect to selecting a more restrictive panel of NED markers to be used [see, e.g., Ref. (12)] and specifying the methods for collecting the NED markers thus is paramount.

The conclusions from these data are: (i) universal NED (SCCP) correlates with poor response to therapy and particularly dim prognosis. Moreover, universal NED includes a large family of tumors that display similar histological, clinical, and prognostic features irrespective of the organ site of origin. This suggests that NED, or at least the extreme end of the NED spectrum, might actually be a rather unique and homogenous disease entity despite its various organ starting points. An in-depth, all-organ-site histological, genetic, phenotypical, and clinical comparative analysis of focal NED is warranted; (ii) focal NED correlates with tumor aggressiveness and poor prognosis, particularly in patients receiving ADT; (iii) CgA is the most reliable immunohistochemical NED marker; (iv) core-needle biopsies provide an accurate snapshot of NED. However, until larger studies are done, surgery remains the gold standard for visualizing NED; (v) serum CgA is the most reliable serum NED marker; and (vi) large studies are needed to help standardize the methodology for detecting and quantifying NED.

## Interleukin-6 and Neuroendocrine Differentiation

Interleukin (IL)-6 plays a key role in ADT-induced NED (63–70, 75). In PCa tissue, IL-6 is found in higher concentrations (albeit more variable in terms of range) than in non-cancerous prostate tissue (69). IL-6 is secreted by several PCa cell lines (PC3, DU145, and TSU), as well as by normal prostatic epithelial cells. By contrast, LNCaP PCa cell line does not secrete IL-6 (66, 69). However, IL-6 receptor is present in all the aforementioned PCa cell lines, including LNCaP (66). Similarly to IL-6, concentrations of IL-6 receptor are higher in PCa, but more heterogeneous in terms of range, as compared to non-cancerous prostate tissue, and its level of expression across PCa samples correlates with increased proliferation (69).

Interleukin-6 induces NED in PCa cell lines, most notably LNCaP, C4-2, and C4-2B (64, 66, 68, 75, 123). The LNCaP cell line provides the model of choice for studying transition of PCa from androgen sensitivity to CR. Because NED and CR in PCa go hand-in-hand (see above), it is not surprising that IL-6 also induces CR. Hobisch’s group was among the first to show that, in principle, IL-6 can activate the AR in an androgen-independent, dose-dependent fashion. The IL-6-activated AR, in turn, activates its target genes at 67% of the level reached in the androgen-activated AR scenario (63). Interestingly enough, IL-6 and androgens have synergistic effects when co-administered in low concentrations, but this additive pattern disappears as IL-6 concentration is increased. The synergistic AR activation by IL-6 and androgens is almost completely inhibited by bicalutamide, an AR blocker (63). In LNCaP cells, IL-6 also activates AR in an androgen-independent fashion (65), increasing AR-regulated PSA gene expression and consequently increasing PSA mRNA and secreted protein levels (63, 70). Co-administration of IL-6 and androgen has a synergistic effect that is blocked by bicalutamide (63, 70). In C4-2 and C4-2B cell lines, IL-6 is a key mediator of bone marrow stroma-induced NED and autophagy (75, 123). Because both NED and autophagy are highly protective for PCa cells, IL-6 might facilitate bone metastasis.

Both NED and CR influence tumor growth. However, the effect of IL-6 on tumor growth remains debatable. In LNCaP cells, several groups showed that IL-6 induces growth (66, 69, 70) while decreasing cell death and proportion of S-phase cells (69). By contrast, others found that IL-6 inhibits LNCaP cell growth via the IL-6 receptor subunit gp130 (64, 67) and decreases proliferation induced by androgens (63). IL-6 inhibits tumor growth by blocking the cell cycle in the G1 phase (64). Cell cycle G1 arrest is associated with an increase in p27^Kip1^ levels and a decrease in CDK2, CDK4, and CDK6 levels (64).

## Signaling Pathways Used by Interleukin-6 to Induce Neuroendocrine Differentiation

The signaling pathways that IL-6 relies on to induce NED and related processes are only partially known and their operation principles are yet to be revealed. It is known that the JAK/STAT3 system, which is a downstream effector of IL-6, is particularly important. Ni and his group found that STAT3 (but not STAT1) is constitutively active in human PCa cell lines (LNCaP, PC3, DU145, TSU) as well as in various Dunning rat PCa sublines (124). Gao’s group reported that, in LNCaP and PC3 cell lines, as well as in PCa samples, STAT3 protein levels are increased twofold over normal prostate tissue (125). In both human and rat PCa cell lines, STAT3 binding activity is correlated with tumor aggressiveness (124). However, other authors report that PC3 cells do not express STAT3 (67).

Giri’s group reported that IL-6 induces phosphorylation and nuclear translocation of STAT3 in both prostatic normal epithelial and LNCaP cells (69). Ueda’s group found that, in LNCaP cells, IL-6 activates the whole JAK/STAT3 system with consequent STAT3 phosphorylation at both sites of regulation (Tyr_705_ and Ser_727_, respectively) (70). In LNCaP cells, IL-6-induced activation of STAT3 triggers NED (66, 68). Furthermore, in PC3 cells, which normally do not express STAT3 (67), overexpression of STAT3 leads to NED (67). Similarly, in C4-2 and C4-2B cells, IL-6 induces NED via activation of STAT3 (75).

In LNCaP cells, JAK/STAT3 signaling also is required for IL-6-induced, AR-mediated gene activation (65). Activated STAT3 associates with AR N-terminal domain (70) in an androgen-independent, IL-6 dependent fashion (65). To this end, phosphorylation, dimerization, and DNA binding of STAT3 (but not STAT1) are crucial. Any one of the AR N-terminal domain regions 234–390 and 391–588 (which STAT3 binds to) can drive the process. By contrast, the 1–233 regions, which do not bind STAT3, are not essential (70). Ueda’s group found that, although JAK plays an important role in general, it is not essential in this specific context (70).

The roles of JAK/STAT3 in NED- and CR-related processes, most notably tumor growth, are still debated. In TSU cells, phosphorylation of STAT3 (via JAK) is required for tumor growth. Thus, overexpression of phosphorylation- and activity-defective STAT3F mutant reduces TSU growth *in vitro*, while decreasing tumorigenicity of TSU cells injected *in vivo* (124). However, Spiotto and Chung reported that TSU line is refractory to STAT3 signaling (or at least to STAT3 signaling induced by IL-6) because of decreased STAT3 tyrosine phosphorylation (67). In LNCaP cells, STAT3 is not essential for cell growth, because wild type and mutant STAT3F cells exhibit similar growth (67). In LNCaP cells, rather than influencing default growth, STAT3 might modulate responses to external signals. Thus, in LNCaP cells expressing wild type STAT3, IL-6 inhibits growth, whereas in LNCaP cells expressing STAT3F mutant, IL-6 enhances growth (67). In PC3 cells, transfected STAT3 inhibits growth (68), while STAT3 knockdown inhibits growth {accompanied by a decrease in c-myc mRNA levels [although see Ref. (67)]}, increases apoptosis (accompanied by a decrease in Bcl-2 protein levels), and induces cell cycle G1 arrest (accompanied by a decrease in cyclin D1 protein levels) (125). In PC3 cells injected *in vivo*, STAT3 knockdown markedly decreases tumorigenic potential and induces intense tumor apoptosis (125). Structurally, the Src homology 2 domain of STAT3 is key in mediating all these effects (125). The DU145 cell line exhibits reduced STAT3 DNA binding, which makes DU145 cells unresponsive to STAT3-mediated IL-6 signaling (67).

It is likely, but yet to be confirmed, that IL-6 participates in more than one signaling pathway to induce these effects. In C4-2 and C4-2B cells, STAT3 activation is required for IL-6-induced NED, but not for IL-6-induced autophagy, which suggests that IL-6 induces these two effects through distinct signaling pathways (75). Besides JAK/STAT3, the other canonical signaling pathways in IL-6 signaling are MAPK and PI3K-Akt. In LNCaP cells, MAPK pathway is required for IL-6-induced (but not for androgen-induced) activation of AR N-terminal domain (70) and consequent expression of AR target genes (63). However, Chen’s group failed to show any such effect in LNCaP cells (65). In IL-6-treated LNCaP cells, the effects of MAPK pathway, if any, do not proceed via JAK/STAT3 phosphorylation; however, some cross-talk is possible as IL-6 induces JAK to phosphorylate MAPK (70). In DU145 cells, MAPK pathway is necessary for IL-6-induced, but not for androgen-induced, activation of AR and expression of the latter’s target genes (63). The PI3K pathway plays a minor role, if any, in IL-6-induced activation of AR N-terminal domain in LNCaP cells (70). Interestingly enough, cAMP and protein kinase A (PKA), as well as protein kinase C, may be involved in IL-6 signaling. In LNCaP cells, inhibition of PKA decreases IL-6-induced, AR-mediated gene expression, while not influencing androgen signaling via AR (63). In DU145 cells, PKA is required for AR signaling induced by either IL-6 or androgens. In DU145, protein kinase C influences AR signaling induced by IL-6 (but not by androgens) (63).

## Correlations between Prostate Cancer and Neural Structures

The significance of NED in PCa is emphasized by the finding that PCa cells benefit from close contact with neural structures. A remarkable attempt to systematize this emerging field was made by Zänker and Entschladen (126). In tissue samples, PCa cells involved in perineural invasion display increased proliferation and decreased apoptosis as compared to PCa cells located away from the nerves (127). Similarly, in an *in vitro* model, first described in Ref. (128), DU145 cells involved in perineural invasion-like actions exhibited increased proliferation and decreased apoptosis, accompanied by an upregulation of several genes and corresponding proteins, three of which (NF-κB, PIM-2, and DAD-1) play anti-apoptotic roles (127). Pharmacologic inhibition of NF-κB reversed most of these effects, as it increases apoptosis and down-regulates NF-κB, PIM-2, and DAD-1 proteins (127). Furthermore, in tissue samples, NF-κB is expressed in higher levels in PCa cells involved in perineural invasion than in corresponding PCa cells remote from the nerves (127). Collectively, these results suggest that NF-κB plays a key role in PCa cells proliferation and survival driven by perineural invasion. Interestingly, high NF-κB nuclear expression in PCa cells exhibiting perineural invasion correlates with recurrence-free survival (127). In addition, PCa cells undergoing perineural invasion upregulate TGFβ1 secretion, which stimulates the perineurium of invaded nerves to secrete caveolin-1. The latter, in turn, inhibits apoptosis in PCa cells (129).

While at least some of the findings above might be the accounted for by the neural stroma, lines of evidence implicate neurons themselves. In mice, prostatic adrenergic sympathetic nerve fibers contribute to the initial stages of PCa development via stromal β_2_- and β_3_-adrenergic receptors, whereas cholinergic parasympathetic nerve fibers play a key role at later stages of tumor invasion, migration, and metastasis through stromal M_1_ muscarinic receptors (130). Interestingly, rather than being skewed in favor of PCa cells, the benefits of perineural invasion might be shared. It has been reported that patients with PCa and preneoplastic lesions have increased global nerve density in the prostate as compared to healthy individuals. Nerve density is higher in tumor foci as compared to non-tumoral regions and healthy prostates, respectively (131), and correlates with increased proliferation of PCa cells and activation of cell survival pathways (including PTEN/Akt-1 and downstream effectors FKHR and GSK, as well as NF-κB and downstream effectors PIM-2 and c-Myc) (132). Furthermore, axonogenesis correlated with aggressive disease and biochemical recurrence (131). In an *in vitro* scenario DU145 cells involved in perineural invasion-like processes upregulated semaphorin 4F gene, which increased neurogenesis (131). Thus, a symbiotic contract might occur between PCa cells and adjacent neural structures (128). One interesting aspect is that not only epithelial but also NE tumor cells can undergo perineural invasion (13). Finally, the Ayala group in collaboration with our group has evidence that PCa cells can adopt a true neural-mimicking phenotype, demonstrated by identification of a subset of “high in brain” expressed genes that also are high in PCa metastases in patients with ADT, and which are proposed to be part of a treatment-resistant phenotype (Farach et al., in preparation). Although they need to be taken *cum grano salis*, these findings beg the question of how a malignant tissue benefits from creating neural-like cells. Because the nervous system is the master device that deals with stress, and ADT is itself a stressful situation, a truly neural differentiation within PCa tissue would seem less far-fetched. But are there any other lines of evidence to support such claims?

The mutual interplay between PCa and neural structures, and the potential advantage of a neural-mimicking PCa phenotype, is supported by the consistent finding that patients with spinal cord injury (SCI) have lower risk of developing PCa than those without SCI (133–136). These findings are consistent with the more general observation that patients with severe SCI have a smaller prostate (133, 137). Several explanations for this phenomenon have been proposed [reviewed in Ref. (135)], mostly related to the disruption of prostatic regulatory neurohormonal axes following the spinal lesion (133, 134). This would account for the observation that risk for PCa is lower only for higher level SCI (above vs. below T6 has been the only cutoff level investigated so far) (135), as prostatic innervation involves lower spinal segments (21). Furthermore, in patients undergoing radical prostatectomy for PCa, general anesthesia (GA) plus neuraxial (spinal or epidural) anesthesia/analgesia (NAA) is associated with lower risk of systemic progression, lower risk of biochemical recurrence, and lower overall mortality as compared to GA plus postoperative opioid analgesia (138, 139). We suggest that this would make sense particularly if one viewed NAA as a temporary, chemical SCI due to the sodium channel blocking action of the agents used, which temporarily interrupts the neural pathways. Inhibition of tumoral voltage-gated sodium channels by non-anesthetic agents inhibits migration, invasion, and metastasis; it has thus been proposed that local anesthetics, due to their main action as voltage-gated sodium channel blockers, might have similar anticancer effects [reviewed in Ref. (140)]. Moreover, cells derived from tumors with universal NED, such as SCLC, generate action potentials relying on inward sodium and outward potassium currents (141, 142). Likewise, normal NE cells in the lung are excitable, rely on voltage-activated potassium, calcium, and sodium currents, and exhibit spontaneous firing modulated by hypoxia [reviewed in Ref. (4)]. In the androgen-independent PCa cell line PC3, growth is inhibited by voltage-gated sodium channel blockers, and the potency of the growth-inhibitory effect is roughly proportional to the potency of the sodium channel-inhibitory effect (143), suggesting that the latter accounts for the growth inhibition.

**Figure 6:**
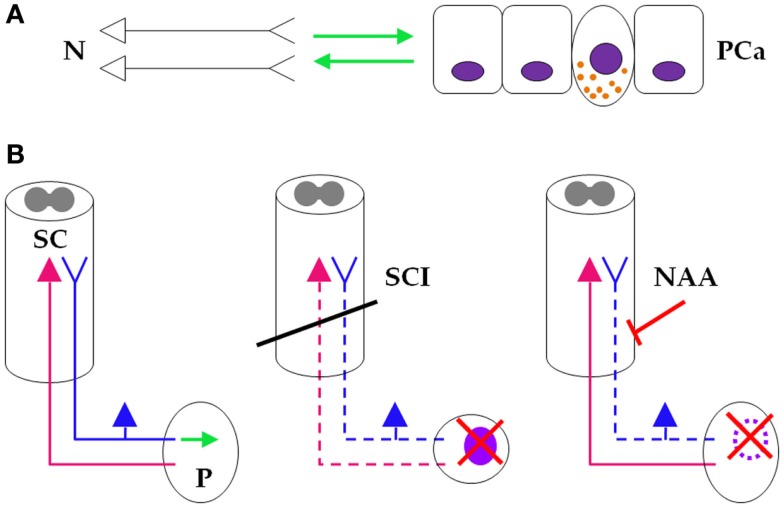
**Neuraxial anesthesia/analgesia as a “temporary spinal cord injury.”** **(A)** At the micro level, neurons *(left)* and prostate cancer cells *(right)* engage in a symbiotic, mutually growth-supportive relationship (green arrows). One NE cancer cell, displaying secretory granules, also is depicted between the non-NE cells. **(B)** *(Left)* At the macro level, sensory prostatic neurons (blue), but not motor prostatic neurons (magenta) support growth of the prostate gland (green arrow). *(Middle)* At the macro level, SCI (oblique black bar) disrupts prostatic innervation (dashed sensory and motor prostatic neurons), which consequently impairs growth and reduces (red saltire) the risk for developing PCa (light purple oval). *(Right)* During PCa surgery, NAA also disrupts prostatic sensory innervation (dashed sensory prostatic neurons) by pharmacologic blockade (oblique red bar-headed line), which reduces risk (red saltire) for recurring PCa (dotted light purple oval line). It is thus reasonable to infer that NAA induces a “temporary SCI” that inhibits PCa similarly to SCI-induced sensory neuronal damage (see text for details). N, neurons; PCa, prostate cancer; SC, spinal cord; P, prostate; SCI, spinal cord injury; NAA, neuraxial anesthesia/analgesia.

One important functional difference between NAA and a real SCI is that the former, in dosages commonly used in the clinic, preferentially targets the sensory nerve fibers while leaving the less susceptible motor fibers relatively unblocked (144). However, it is the sensory, and not the motor innervation of the prostate, which is responsible for neural regulation and support of prostate growth and development (21). It is thus tempting to construct a unified hypothesis, in which the PCa/nerve symbiosis accounts for the reduced risk for PCa in patients with damaged prostatic innervation following SCI, which further explains the reduced risk for PCa recurrence in PCa patients with temporarily damaged prostatic innervation following NAA (Figure 6). Attempts to target PCa with neurotropic agents that block the generation or transmission of action potentials are thus legitimate. Botulinum toxin has been shown to inhibit the growth of LNCaP cells *in vitro* and *in vivo* (145), and an ongoing clinical trial is investigating the effects of botulinum toxin on PCa (see NCT01520441 on www.clinicaltrials.gov). Before pursuing this lead, however, one should first determine if the observed effects of NAA might be better accounted for by non-neurotropic factors.

It is widely assumed that most general (as opposed to neuraxial) anesthetics are immunosuppressive, mainly by decreasing natural killer (NK) cell activity, and promote cancer metastasis [Ref. (146); also reviewed in Ref. (140)]. NAA might help avoid this immunosuppression by reducing the use of GA [reviewed in Ref. (140)]. However, several studies found that patients receiving both GA and NAA had better outcomes in terms of PCa biochemical recurrence (138), local recurrence (147), systemic progression (metastasis) (139, 147), and/or mortality (139), or in terms of colorectal cancer mortality (148) than patients receiving GA alone. Because GA was given to both study arms, it follows that in all these cases NAA did not help avoid the use of GA. If the only anticancer effect of NAA was to help avoid GA, then no significant differences in terms of immunosuppression should have been present between the arms. Still, significant differences in outcome were present. Thus, it follows that NAA might act through additional undiscovered mechanisms.

Opioid agents, which are routinely used for surgical analgesia, are immunosuppressive [reviewed in Ref. (140)]. Another proposed mechanism by which NAA decreases perioperative immunosuppression is through reducing the need for systemic opioids [reviewed in Ref. (140)]. Sprung’s group performed two significant retrospective studies that showed that opioids can be important confounders. In patients undergoing radical prostatectomy for PCa, GA plus NAA lowered risk of systemic progression and overall mortality as compared to GA plus postoperative opioid analgesia (139). Interestingly, no significant differences were found when NAA scheme included fentanyl (149), which is rapidly absorbed into the systemic circulation due to its high liposolubility [reviewed in Ref. (149)]. The beneficial effects of NAA, including the decreased need for systemic opioids, might have been neutralized by the immunosuppressive effects of fentanyl escape into the bloodstream (149). The same should in principle apply to the other lipophilic opioids (e.g., sufentanil), which might explain why studies in which the NAA patients received intraneuraxial lipophilic opioids (150–153) found no significant differences between the two anesthetic techniques (149). The other studies reporting non-significant or equivocal differences did not mention the pharmacologic agents used (147, 154–156).

It is expected that NAA works through both mechanisms described above, i.e., helping avoid both GA and systemic opioids [reviewed in Ref. (140)]. However, a careful inspection reveals that both mechanisms rely on the *passive avoidance* of an *extrinsic* pharmacological effect of some other agent, rather than the *active promotion* of an *intrinsic* pharmacological effect of their own. In other words, NAA is devoid of antitumor effect *per se*, which has actually been proposed (149). This being the case, adjusting for the corresponding confounders, i.e., presence of GA in both arms under study and intraneuraxial injection of lipophilic opioids in patients receiving NAA, should render uniform results across various cancer types. However, the results indicate that this is not the case. In patients undergoing surgery for non-metastatic rectal, as opposed to colon cancer, GA plus NAA followed by postoperative NAA were associated with reduced mortality as compared to GA alone followed by postoperative opioid analgesia (148). The fact that the NAA arm had better outcomes in rectal cancer patients is unexplainable, as no significant differences were found between the two cancer type groups (rectal vs. colon) with respect to the inclusion of opioids or the inclusion of lipophilic opioids in the NAA regimen, and GA was used in both therapeutic arms for both cancer type groups (148). Therefore, any confounder determined by the rapid absorption of neuraxially administered fentanyl or sufentanil into the systemic circulation, as well as any confounder related to GA use in both arms, should have manifested itself uniformly across the two cancer types. Besides that, opioids do not have significant immunosuppressive effects in colorectal cancer. Patients with colorectal cancer display suppression of NK cell activity, which has important prognostic significance and is reversed following tumor resection [reviewed in Ref. (157)]. However, opioids given in analgesic concentrations do not induce NK cell-mediated immunity in treated patients (158). In fact, some opioids, including fentanyl, might increase NK cell activity [reviewed in Ref. (158)] or even have antitumor effects on colon cancer *in vivo* [reviewed in Ref. (159)]. Given these findings, we suggest that a plausible explanation for the observed discrepancies between rectal and colon cancer patients would be that NAA has a pharmacological antitumor effect *per se*. In other words, this is not a merely *passive* antitumor effect due to *avoiding immunosuppressive factors*, but rather an *active* antitumor effect, either due to *inhibiting immunosuppressive factors* and/or to *promoting* or *inhibiting non-immunological factors*. Such an intrinsic pharmacologic action could explain why NAA yields better outcomes in some, rather than all, cancer types, including prostate (138, 139), breast (160), hepatocellular (161), rectal (148), ovarian (162), and melanoma (163); also see Ref. (164) and the references therein.

Obviously, this simple arithmetic cannot cover all the details. It is difficult to speculate upon the nature of the active anti-immunosuppressive effect of NAA, if any, mostly because it has become increasingly clear that cancer-associated immune response and immunosuppression are tumor type-specific and correlate with disease staging, therapy, and prognosis [see Ref.(157, 165, 166) and the references therein]. In particular, this holds for PCa (167). However, it has been suggested that NAA decreases perioperative immunosuppression through blocking sympathetic activation and decreasing plasma levels of catecholamines and cortisol [reviewed in Ref. (140)]. These are genuine neurotropic effects, which makes the neurotropic-based antitumor effect of NAA plausible.

In the same vein, it remains to be seen if NAA has active non-immunologic, neurotropic anti-PCa cell growth effects. A recent meta-analysis found no significant differences between the effect of NAA and GA, respectively, on postoperative function of NK T lymphocytes (168). Since NK T lymphocytes are crucial for anticancer immunity [reviewed in Ref. (168)], it might be that NAA does not differ from GA in terms of immunosuppression. In this case, the beneficial effects of NAA on cancer outcomes as compared to GA might be better explained by active non-immunologic effects of the former rather than by active immunologic effects. Because the antitumor neurotropic effect, be it immunologic or non-immunologic, seems the most plausible scenario, we argue that it is legitimate to start by seeking non-immunologic effects of NAA on PCa that are also neurotropic in nature, most notably a disruption of the PCa/neural symbiosis.

## Correlations between Prostate Cancer and Neural Functions

In addition to these associations between PCa and peripheral neural structures, a growing body of evidence indicates an existing association between PCa and central neural functions. This, too, might shed light upon the significance of processes such as NED. Following almost a century of observations, systematic nationwide studies conducted during the last three decades have revealed an intriguing association between schizophrenia and a reduced risk for cancer as compared to the general population (169–175), although two other nations reported no difference or even an increased risk (176, 177). The reduced risk is more pervasive in males (169, 170, 172, 174, 175), and the risk reduction most consistently reported is for PCa [Ref.(169, 171–175); a similar, yet not significant trend was also reported in Ref. (178)]. The mechanisms responsible for these findings are incompletely understood, but use of antipsychotic drugs, which are used in several psychiatric disorders, including schizophrenia, correlates with reduced risk of developing cancer (179, 180), most notably PCa (179). A protective genetic trait cannot be ruled out, though, because some instances of cancer risk reduction in schizophrenia had first been mentioned in 1909 [reviewed in Ref. (177)], that is, some 45 years before the discovery of antipsychotics (181). A meta-analysis reported large sets of genes and pathways that are dysregulated in opposite directions between three CNS disorders (schizophrenia, Parkinson’s, and Alzheimer’s disease) and three cancers (PCa, lung, and colorectal cancer), either by being upregulated in one or more of the three CNS disorders and downregulated in one or more of the three cancers, or vice versa (182). On the other hand, the up- or downregulation of at least some of these genes and pathways might be medication-related rather than owed to the disease itself. This leaves open the possibility that some of the drugs used in CNS disorders might downregulate certain oncogenes, or upregulate certain tumor suppressor genes, thus conferring anticancer protection (182). Furthermore, parents with schizophrenic offspring have the same risk for developing cancer as parents with no schizophrenic offspring [Ref. (183), although see Ref. (174, 176), but in these studies the controls were general population rather than parents with healthy offspring]. This suggests that it is a non-shared, environmental, rather than a shared, inherited factor, which accounts for most anticancer protection in schizophrenics, which further supports the antipsychotic medication hypothesis. Among the antipsychotics currently used, phenothiazine compounds are best documented to associate with decreased risk for cancer, most notably PCa (179).

Experimental findings lend further support to these clinical findings. *In vitro*, trifluoperazine, a phenothiazine antipsychotic agent, decreases proliferation, induces depletion of cancer stem cells (CSCs), and overcomes gefitinib resistance in non-small cell lung cancer (NSCLC) cell lines (184). Similarly, thioridazine, another phenothiazine antipsychotic agent, selectively induces differentiation (i.e., loss of pluripotency) of human CSCs both *in vitro* and *in vivo* (namely, acute myeloid leukemia stem cells) while sparing normal stem cells, and allows for 100-fold dosage reduction of the antileukemic drug cytarabine when co-administered with it (185). Moreover, the mechanism of action involves mainly D_2_ dopamine receptors, which also account for the antipsychotic effects of the agent (181, 186). Further extending the screening pool identified two additional phenothiazine agents (namely, prochlorperazine and fluphenazine) as having similar effects on CSCs *in vitro*. Although the pharmacologic effects of these additional two agents were weaker and less selective as compared to thioridazine (185), the data collectively raise the interesting possibility that the effect on CSCs might be a class effect (187).

## Interleukin-6 is a Common Denominator in the Pathogenesis of Neuroendocrine Differentiation and Schizophrenia

Apart from playing a central role in ADT-induced NED, IL-6 has recently come to attention as a key player in the pathogenesis of schizophrenia. The evidence for this is fourfold.

First, patients with schizophrenia (especially those previously untreated or not receiving medication for prolonged periods of time) have high plasma levels of IL-6 compared to healthy controls (188–191), and the increased levels correlate with acute phase (192). However, antipsychotic treatment decreases plasma levels of IL-6 and soluble IL-6 receptor (188, 189). Plasma levels of IL-6 also are increased in elderly schizophrenics with long-term disease and persisting symptoms despite long-term therapy, which could reflect resistance to therapy and thus explain the apparent paradox (193).

Second, in middle-aged adults, plasma levels of IL-6 correlate inversely with the volume of hippocampal gray matter (194). The hippocampus is an important brain region linked to the anatomic basis of schizophrenia [reviewed in Ref. (195)], and hippocampal volume is decreased in schizophrenic patients (191).

Third, IL-6 gene polymorphism is associated with schizophrenia [Ref. (190), although see Ref. (196)] and reduced hippocampal volume occurs in antipsychotic-naïve schizophrenic patients (191).

Fourth, IL-6 is a key player in established pathogenic models of schizophrenia, including the “ketamine model” (197). Namely, IL-6 acts as a key downstream effector of the NMDA receptor antagonist ketamine that activates NADPH oxidase in the brain, leading to increased superoxide production and consequent dysfunction of parvalbumin-expressing inhibitory interneurons (197). The dysfunction of these GABA-ergic interneurons, in turn, has been linked to the pathogenesis of schizophrenia [reviewed in Ref. (197)].

## Concluding Remarks: Seeking Neural Differentiation in Prostate Cancer

It is commonly accepted that PCa NED is enrichment of a cell subset that secretes various neuropeptides leading to CR of PCa [see, e.g., Ref. (49)]. It is thus not surprising that PCa NED most frequently appears following ADT and therefore acts as an “escape” mechanism whereby advanced PCa can evade current therapeutic strategies. A crucial molecular mediator is IL-6, which acts as a signaling bridge linking ADT, via STAT3 and activation of AR target genes in the absence of androgens, to the ensuing NED and CR.

Prostate cancer also displays profound correlations with neural structures: (i) at the sub-micro level, PCa cells express many genes expressed in neurons and other CNS cells; (ii) at the micro level, PCa and neurons engage in a symbiotic-like relationship; (iii) at the macro level, SCI reduces risk for PCa [which further explains (ii)]; (iv) at the macro level, NAA, but not GA, reduces risk for PCa recurrence by acting as a “temporary SCI” through blocking the action potentials of sensory neurons. The NED cancers also exhibit action potentials [which further supports (ii) and (iii)].

Prostate cancer also is associated intimately with neural functions: (iv) schizophrenia decreases risk of cancer, most notably PCa; (v) antipsychotics explain this risk reduction; (vi) at the micro level, antipsychotics exhibit strong anticancer activity, including strong effects on CSCs; (vii) IL-6, the *prima donna* of ADT-induced NED, is a key player in the pathogenesis of schizophrenia. Its high levels encountered in these patients are decreased by antipsychotics. If corroborated to (iv), (v), and (vi), this might provide a further connecting lead to the effects of IL-6 in NED.

We propose that the lines of evidence (i)–(vii) make it reasonable to think that there is a real neural trait in PCa, and most notably in NED PCa, that should better be explored mechanistically. The phenomenon of cancer displaying neural differentiation has been previously reported for melanoma [see Ref. (198, 199) and the references therein]. Along the same chain of reasoning, brain metastases of both HER2 and triple-negative breast cancer have recently been found to switch to a neuronal- and glial-like GABA-ergic phenotype as compared to their primary tumor counterparts (200). However, both melanocytes and mammary epithelial cells have a completely different developmental origin from normal and PCa NE cells [reviewed in Ref. (198, 199, 201)]. True neural differentiation of PCa, if properly explored, would thus open a fascinating view onto the biology of malignant tumors undergoing NED, neural differentiation, or a mixture of the two.

## Conflict of Interest Statement

The authors declare that the research was conducted in the absence of any commercial or financial relationships that could be construed as a potential conflict of interest.

## Abbreviations

ADT, androgen deprivation therapy; AR, androgen receptor; BPH, benign prostatic hyperplasia; CgA, chromogranin A; CR, castrate resistance; CSC, cancer stem cells; DAD-1, defender against apoptotic cell death 1; EPSCC, extrapulmonary small cell carcinoma; GA, general anesthesia; IL, interleukin; JAK, Janus kinase; MAPK, mitogen-activated protein kinase; NAA, neuraxial anesthesia/analgesia; NE, neuroendocrine; NED, neuroendocrine differentiation; NF-κB, nuclear factor kappa B; NK, natural killer; NSE, neuron-specific enolase; PCa, prostate cancer; PI3K, phosphoinositide 3-kinase; PIM-2, provirus integration of Moloney virus 2; PKA, protein kinase A; SCCP, small cell carcinoma of the prostate; SCLC, small cell lung carcinoma; SCI, spinal cord injury; STAT, signal transducer and activator of transcription; VEGF, vascular endothelial growth factor.
